# One-size-fits-all strategy in carotid artery treatment using CGuard stent, feasibility and clinical pilot study

**DOI:** 10.1186/s42155-025-00601-7

**Published:** 2025-10-16

**Authors:** Simone Balocco, Juan Rigla, Reimer Andresen, Christian Wissgott

**Affiliations:** 1 Department of Mathematics and Computer Science, University of Barcelona, Gran Via 585, Barcelona, 08007 Spain; 2 Computer Vision Center, Bellaterra, 08193 Spain; 3 Inspire M.D, 6303 Waterford District, Miami, FL 33126 USA; 4 Institute of Diagnostic and Interventional Radiology/Neuroradiology, Westküstenklinikum Heide, Esmarchstraße 50/Haus C, Heide, 25746 Germany; 5 Institute for Diagnostic and Interventional Radiology/Neuroradiology, Schön Klinik Rendsburg, Lilienstraße 20-28, Rendsburg, 24768 Germany; 6 Hospital of the Universities of Kiel, Hamburg and Lübeck, Arnold-Heller-Str 3, Kiel, 24105 Germany

**Keywords:** Carotid artery stenting, Nitinol, One-size-fits-all, Conformability, CGuard

## Abstract

**Purpose:**

Conventionally, the treatment of carotid artery disease customizes stents to the vessel diameter. The design of the CGuard stent and its nitinol shape memory allow for a wide operational range and minimal residual radial force. This pilot study assesses the feasibility and clinical outcomes of the 10-mm CGuard stent in a one-size-fits-all sizes protocol.

**Materials and methods:**

The study is a multicenter, prospective cohort study involving 226 consecutive patients with symptomatic or asymptomatic carotid artery stenosis, with an indication for revascularization. All patients received the 10-mm CGuard stent, irrespective of the carotid artery reference diameter, and were grouped into three categories based on the reference vessel diameter. Endpoints included procedural success and incidence of major adverse cardiovascular events (MACE) at 30 days and 1 year.

**Results:**

The study achieved 100% technical success, with an average residual stenosis of 8.41%. Clinical success was 98.7%. During in-hospital observation, one patient experienced a major ipsilateral stroke (0.4%), and two patients had transient ischemic attacks (TIAs) (0.8%). At the 30-day follow-up, there were one death and one case of re-occlusion which was asymptomatic. No differences between the three groups were found regarding technical or procedural success, residual stenosis, complications, TIAs, MACEs, or patency rates. No events occurred between the 30-day and 1-year follow-up, maintaining the MACE rate at 1.5%. ICA and ECA patency rates at 1 year were 99% and 99.4%, respectively, indicating mid-term treatment effectiveness.

**Conclusion:**

This pilot study demonstrates that the one-size-fits-all approach using a 10-mm CGuard stent to treat carotid artery stenosis provides minimal residual radial force with optimal stent apposition. The one-size-fits-all approach with CGuard stent is feasible, safe, and effective. Further studies for confirmation are guaranteed.

**Supplementary Information:**

The online version contains supplementary material available at 10.1186/s42155-025-00601-7.

## Clinical impact

The use of a single-size 10-mm CGuard nitinol stent appears feasible, safe, and effective for the treatment of carotid artery stenosis during 1-year follow-up.

The additional benefit of the one-size-fits-all strategy would be:

- To guarantee optimal vessel apposition to the vessel wall with minimal residual radial force
- To streamline stent sizing and reduce inventory storage needs

## Introduction

### Clinical background/motivation of the study

Carotid artery disease has been related to stroke risk in asymptomatic and symptomatic patients over the mid-term [1]. In recent years, second-generation (mesh-covered) carotid stents improved the carotid endovascular revascularization results [2, 3].

Additionally, new studies have demonstrated the potential to enhance the usability of the CGuard stent according to the “one-size-fits-all.” Such strategy suggests that a single nitinol stent size (10 mm) can adapt to multiple target artery diameters [4, 5].

In current clinical practice, when a conventional stent is used, the stent size is usually chosen to closely match the maximum diameter of the vessel to be treated, as recommended by CIRSE Standards [6]. For example, for a 6-mm vessel, a 7-mm stent is typically selected. Instead, in this pilot study, it is proposed to employ exclusively 10-mm diameter CGuard stents to treat three groups of patients with different common carotid artery reference diameters (6 to 7 mm, > 7 to 8 mm, and > 8 to 9 mm) and to compare the clinical outcomes of these three groups.

The one-size-fits-all strategy is specifically facilitated by the use of a nitinol CGuard stent, which adapts to varying diameters due to its design, which exploits the properties of nitinol. The potential benefit of the one-size-fits-all strategy would be to ensure optimal vessel apposition to the vessel wall while also streamlining stent sizing and reducing inventory storage needs.

Therefore, the present pilot study aims to evaluate the feasibility of the one-size-fits-all strategy in a clinical pilot study, in which a nitinol CGuard mesh-covered stent is applied to unselected consecutive patients of carotid artery disease.

### Nitinol stent properties

The challenge of the ideal stent design to dilate the stenotic segments with strong elastic force and conform the larger healthy segments with a lower residual force.

**Fig. 1:**
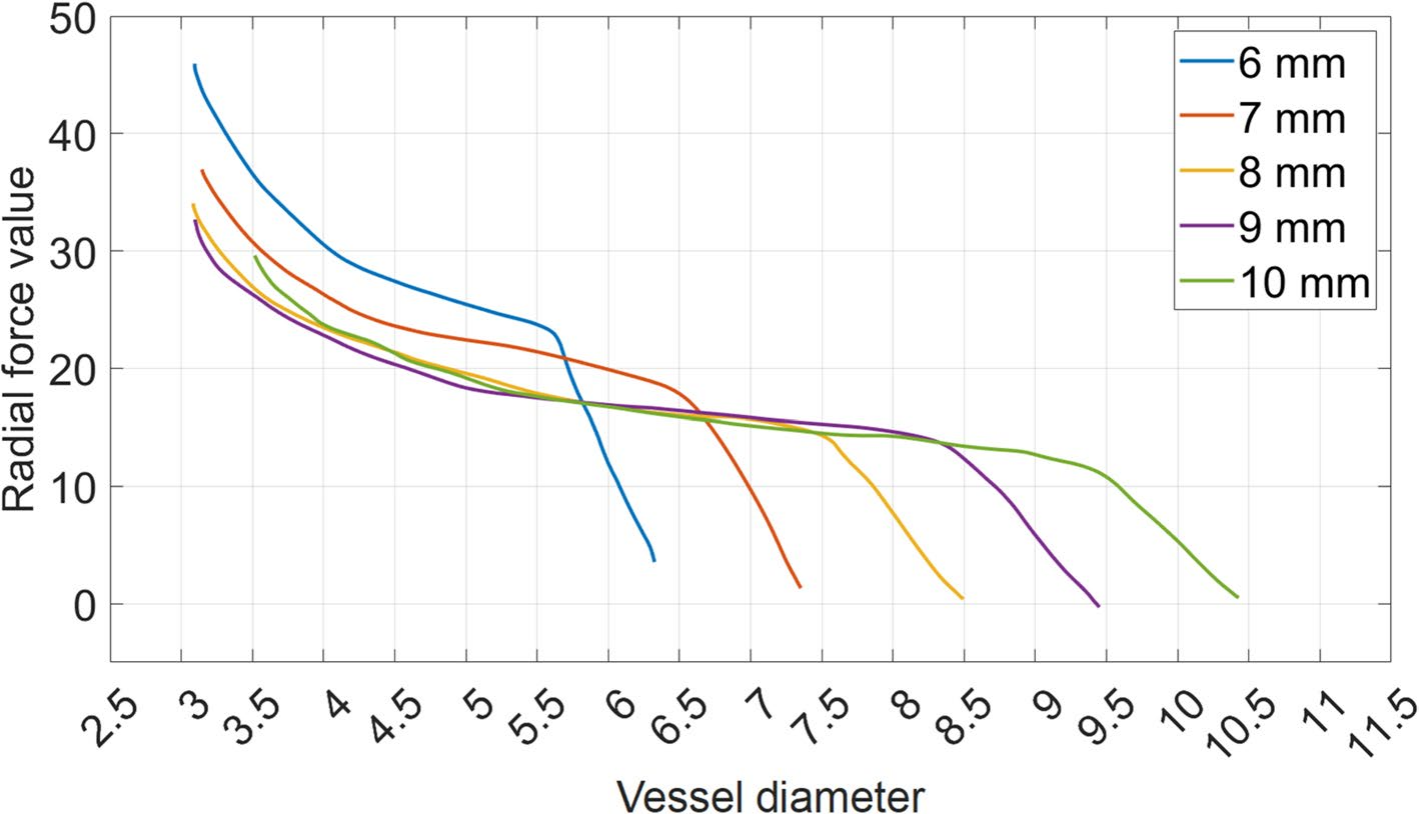
Diagram of residual radial force value as function of the vessel diameter for CGuard stent. The curve of each stent size is depicted in a different color (namely 6 mm in blue, 7 mm in red, 8 mm is yellow, 9 mm is magenta, and 10 mm is green)

Nitinol alloys exhibit two closely related properties: the “superelasticity” and the “shape memory” effect [7, 8]. The combination of such effects define the hysteresis curves shown in Fig. 1 [9].

- “Superelasticity” is the ability of the metal to undergo large deformations and immediately return to its undeformed shape upon removal of the external compression.
- “Shape memory” refers to nitinol’s ability to revert to a specific shape at a designated “transformation temperature.” It gradually returns to its thermally pre-defined shape and diameter at body temperature (≥ 37 °C).

Carotid nitinol stents are build using a tube 5 mm large in diameter. When the stent is delivered, it exhibits its “superelasticity.” The radial force is proportional to the degree of deformation from the original 5-mm diameter tube frame, behaving like a spring.

Additionally, the nitinol stent is thermally pre-deformed to its nominal stent size (in the case of the 10-mm stent size). The “shape memory” effect in the stent activates upon reaching body temperature, gradually expanding the stent from 5 to 10 mm to match the vessel diameter with minimal residual force.

In this manner, during the stent release the predominant force is “superelasticity” strength, while during apposition phase the force of “shape memory” predominates, allowing the stent to adapt to the vessel’s shape for optimal adaptability.

### One-size-fits-all strategy

When a conventional stent is used, the stent size is typically chosen considering the vessel to be treated. For instance, for a 6-mm vessel, a 7-mm stent is generally selected. However, the ideal stent selection should also consider two additional criteria: the stent “working range” and the “residual radial force.”

A stent’s “working range” relies on its capacity to accommodate varying diameters. For instance, the 6.0-mm CGuard stent is suitable only for vessels up to 5.0 mm wide (Fig. 1). However, its radial force diminishes rapidly with larger diameters, restricting its use above 6 mm. In contrast, a 10-mm stent accommodates vessels up to 9.5 mm while maintaining an appropriate force between 4.5 and 9.5 mm.

The term stent “residual radial force” refers to the force exerted by the stent on the vessel wall. Excessive residual radial force can cause injuries. The optimal stent should apply a minimal residual radial force at any diameter. In the case of a 10-mm stent, the magnitude of the residual radial force is lower than the one produced by smaller diameter stent sizes and it is maintained across the stent’s entire working range.

Figure 1 illustrates the CGuard stent expansion radial force values, with overlapping curves for different stent sizes. As can be seen, the CGuard 10-mm stent, thanks to the nitinol material’s shape memory property, meets both requirements by providing the widest working range (4.5–9.5 mm) and the lowest residual radial forces across all diameters through a gradual application of radial force until complete apposition.

Based on these premises, we conducted this study using exclusively a 10-mm diameter CGuard stent for any vessel stent diameter, exploring the one-size-fits-all principal strategy.

### Novelty of the study

Two previous studies laid the groundwork for the current research. In the first study [4], the mechanical properties of various sizes of CGuard stents were examined, and a series of 30 patients were treated with different stent sizes. In the second study [5], for the first time, the one-size-fits-all strategy with a 10-mm stent was tested in 30 unselected patients analyzed at 30 days.

This novel research aims to validate the clinical outcomes of the one-size-fits-all strategy in a larger cohort of 226 patients, with a 1-year follow-up period.

## Method

The study was designed as a multi-center prospective cohort study of consecutive patients with criteria for carotid endovascular revascularization, exclusively using a one-size-fits-all strategy with the CGuard 10 mm size diameter as the sole study device.

### Materials

Inclusion criteria for this study include adults aged ≥ 19 years with carotid artery disease suitable for carotid artery stenting, willingness and ability to consent, compliance with protocol requirements and at least 30 days of dual antiplatelet therapy, life expectancy of ≥ 24 months, modified Rankin score ≤ 2, and non-pregnant/non-lactating females. Participants must have symptomatic stenosis ≥ 50% or asymptomatic stenosis ≥ 80%, alongside medical or anatomical high-risk factors for carotid endarterectomy, such as advanced age, significant cardiac or pulmonary conditions, prior neck surgeries or radiation, anatomical challenges, or planned major vascular procedures 31–60 days post-stenting. Exclusion criteria include recent or upcoming vascular interventions (within 30 days), severe anatomical constraints, total vessel occlusion, severe kidney impairment, bleeding or clotting disorders, specific allergies, previous stenting, dementia, or clinical conditions making intervention hazardous.

The principal endpoints were procedural success and incidence of major adverse cardiovascular events (MACE) at 30 days and 1 year. MACEs are defined as stroke, death, and myocardial infarction.

This study was approved by the Institutional Review Board (IRB). The patient signed an informed consent to be included in this study. The protocol was executed in two centers specialized in interventional radiology with a high incidence of symptomatic carotid patients, including ongoing stroke cases. The study follows the applicable guidelines and treatment recommendations and the center protocols of carotid stenting.

The study included patients requiring urgent revascularization as well as those undergoing elective procedures. The surgical option was considered only when the intervention was deemed contraindicated. All cases were performed by experienced operators, and follow-up included neurological assessments and Doppler evaluations.

In some cases, the follow-up was interrupted by the COVID pandemic policies. However, no patient was dismissed before at least three attempts to telephone for follow-up. Mid-term treatment consisted of aspirin for life according to protocol and clopidogrel for at least 3 months after the initial treatment. Management of dyslipidemia and hypertension was established to ensure the best treatment approach for each patient.

### Statistics

The statistical analysis performed in this paper is computed as follows: continuous data are summarized in terms of mean, standard deviation (SD), and median. Categorical data are summarized in terms of the number of cases (*n*), frequency counts, and percentages. Percentages are presented with one decimal place. The statistical expert performing the computations was blinded to each stent brand’s product vendor to ensure unbiased analysis. The sample size was calculated to ensure adequate statistical power to detect a clinically meaningful effect and under the hypothesis of a patient drop of 15%.

One-way ANOVA analysis was performed within and between-group comparisons for continuous data. Chi-square tests were performed for categorical data analysis. A *p* value inferior to 0.05 has been considered statistically significant in all the statistical tests. The results are qualitatively illustrated by means of boxplots.

## Results

### Patient cohorts

From 2019 to 2023, we recruited 226 consecutive patients with either symptomatic or asymptomatic carotid stenotic disease (68.1% male) who were undergoing elective or urgent revascularization, with or without an ongoing stroke. Two experienced interventional radiologists (clinician 1 and clinician 2) performed the interventions in two interventional radiology departments (Schön Klinik Rendsburg and Westküstenklinikum Heide). Of the 226 patients, 65 were treated by clinician 1, while 161 were treated by clinician 2.

Most of the patients were symptomatic (80.9%) and 21.2% were affected by atrial fibrillation, 41.5% presented a recent TIA < 24 h, and 33% had an ongoing stroke associated to the carotid disease (tandem lesion). 10.1% of the subjects had a ranking modified scale of 3 or 4 at arrival (see Table 1). The patient characteristics were homogeneous, along the three reference diameter classes, except for the two patients included with antecedents of radiotherapy, which presented only diameters of class 1.

**Table 1:** Patient characteristics divided in CCA reference diameter class 1 [6–7) mm, class 2 [7–8) mm, and class 3 [8–9) mm

Title	Items	All	(All %)	Class1	(Class1%)	Class2	(Class2%)	Class3	(Class3%)	*p*-val
*N*		226		81		124		21		
Age	Mean (± std)	70.9	(± 9.17)	70.69	(± 8.74)	71.1	(± 9.48)	70.57	(± 9.28)	0.9373
	Median	71.5		71		72		70		
Gender	Female	72	(31.8%)	23	(28.3%)	42	(33.8%)	7	(33.3%)	0.47157
	Male	154	(68.1%)	58	(71.6%)	82	(66.1%)	14	(66.6%)	
Symptomatic for cerebral ischemia	Asymptomatic	43	(19%)	12	(14.8%)	25	(20.1%)	6	(28.5%)	0.13832
	Symptomatic	183	(80.9%)	69	(85.1%)	99	(79.8%)	15	(71.4%)	
Diabetes	Yes	106	(46.9%)	40	(49.3%)	56	(45.1%)	10	(47.6%)	0.68804
Dyslipidemia	Yes	107	(47.3%)	34	(41.9%)	63	(50.8%)	10	(47.6%)	0.34114
Smoker	Yes	144	(63.7%)	53	(65.4%)	77	(62%)	14	(66.6%)	0.863
Hypertension	Yes	184	(81.4%)	64	(79%)	101	(81.4%)	19	(90.4%)	0.2832
Coronary artery disease	Yes	122	(53.9%)	46	(56.7%)	63	(50.8%)	13	(61.9%)	0.89495
AF (atrial fibrillation)	Yes	48	(21.2%)	14	(17.2%)	30	(24.1%)	4	(19%)	0.47062
Carotid radiotherapy	Yes	2	(0.8%)	2	(2.4%)	0	(0%)	0	(0%)	0.041971
Previous CEA (carotid endarterectomy)	Yes	4	(1.7%)	1	(1.2%)	3	(2.4%)	0	(0%)	0.95965
Previous CAS (carotid arterial stent)	Yes	3	(1.3%)	2	(2.4%)	0	(0%)	1	(4.7%)	0.84726
Previous stroke	Yes	81	(35.8%)	27	(33.3%)	50	(40.3%)	4	(19%)	0.73658
Minor stroke	Yes	57	(25.2%)	20	(24.6%)	34	(27.4%)	3	(14.2%)	0.6425
Major stroke	Yes	22	(9.7%)	7	(8.6%)	13	(10.4%)	2	(9.5%)	0.76031
Basilar stroke	Yes	0	(0%)	0	(0%)	0	(0%)	0	(0%)	1
Ongoing stroke	Yes	75	(33.1%)	26	(32%)	45	(36.2%)	4	(19%)	0.63221
Retinian	Yes	25	(11%)	7	(8.6%)	13	(10.4%)	5	(23.8%)	0.11326
Ranking modified scale	0	147	(65%)	54	(66.6%)	77	(62%)	16	(76.1%)	0.81642
	1	21	(9.2%)	5	(6.1%)	15	(12%)	1	(4.7%)	
	2	35	(15.4%)	14	(17.2%)	19	(15.3%)	2	(9.5%)	
	3	19	(8.4%)	7	(8.6%)	10	(8%)	2	(9.5%)	
	4	4	(1.7%)	1	(1.2%)	3	(2.4%)	0	(0%)	
Previous TIA < 24 h (transient ischemic attack)	Yes	94	(41.5%)	40	(49.3%)	46	(37%)	8	(38%)	0.12212

Basal stenosis was 78.99 (± 8.71). The left internal carotid artery (LICA) was involved in 50.4% of the cases, while the right internal carotid artery (RICA) was involved in 49.6% of the cases. Premedication with aspirin and clopidogrel was administered to all the patients, and when doses were missing, charged doses were applied. IV heparin unfractionated was administered at 100 IU/kg, adjusted according to ACT coagulation tests superior to 250 s.

A distal embolic protection device was used in 15.9% of cases, based on the operator’s decision, which considered patient risks as well as lesion and plaque characteristics. Among the patients in which the embolic protection was used, 27.7% were ulcerated, 58.3% were calcified, and 38.8% were thrombotic.

The study protocol was designed for using only CGuard carotid stent 10 mm diameter for all the reference diameters (class 1, 2, and 3) according to a strategy one-size-fits-all. In all cases, the 10-mm CGuard stent was implanted. The length distribution of the implanted stents was 76.9% with 10 mm × 40 mm and 23% with 10 mm × 30 mm. From the total study patient cohorts, 12.3% were pre-dilated and 100% were post-dilated with a 5.0 to 6.0 balloon at 10 ATM. Patient characteristics are detailed in Table 1. Procedural characteristics show no statistical differences among groups.

In order to compare the clinical outcomes of three patient cohorts, the reference diameters were segmented according to the CCA diameter in three groups: class 1 (6 ≤ x < 7 mm, namely [6–7) mm), class 2 (7 ≤ x < 8 mm, namely [7–8) mm), and class 3 (8 ≤ x < 9 mm, namely [8–9) mm).

### Procedural characteristics

Symptomatic patients undergoing CAS represent a known post-procedural risk group for MACEs. The complete list of cerebrovascular risk factors is presented in Table 2 according to class distributions. As shown in Table 2, no statistically significant differences were found among the risk variables, as indicated by their non-significant *p* values, ensuring that the groups are balanced is crucial to discard any bias in clinical outcome differences due to variations in the patient cohort. Therefore, in Tables 5 and 6, we focused on analyzing independent risk factors [3] for post-procedural events extracted from Tables 1 and 2.

**Table 2:** Procedural characteristics divided in CCA reference diameter class 1 [6–7) mm, class 2 [7–8) mm, and class 3 [8–9) mm

Title	Items	All	(All %)	Class1	(Class1%)	Class2	(Class2%)	Class3	(Class3%)	*p*-val
*N*		226		81		124		21		
Internal carotid artery side	LICA	114	(50.5%)	42	(51.8%)	61	(49.1%)	11	(52.3%)	0.87418
	RICA	112	(49.5%)	39	(48.1%)	63	(50.8%)	10	(47.6%)	
Stent brand	CGuard	226	(100%)	81	(100%)	124	(100%)	21	(100%)	1
Stent 1 size	10 × 30	52	(23%)	19	(23.4%)	31	(25%)	2	(9.5%)	0.41162
	10 × 40	174	(76.9%)	62	(76.5%)	93	(75%)	19	(90.4%)	
Stenosis pre (percentage)	Mean (± std)	78.99	(± 8.71)	79.49	(± 8.3)	79.4	(± 8.64)	74.61	(± 9.88)	0.053615
	Median	80		79		80		75		
Culpit ICA reference diameter	6	139	(61.5%)	79	(97.5%)	59	(47.5%)	1	(4.7%)	4.5082e − 24
	7	81	(35.8%)	2	(2.4%)	65	(52.4%)	14	(66.6%)	
	8	6	(2.6%)	0	(0%)	0	(0%)	6	(28.5%)	
Culpit CCA reference diameter	7	81	(35.8%)	81	(100%)	0	(0%)	0	(0%)	4.1953e − 66
	8	124	(54.8%)	0	(0%)	124	(100%)	0	(0%)	
	9	21	(9.2%)	0	(0%)	0	(0%)	21	(100%)	
AAS (ASPIRINE) premedication	Yes	226	(100%)	81	(100%)	124	(100%)	21	(100%)	1
Clopidogrel premedication	Yes	226	(100%)	81	(100%)	124	(100%)	21	(100%)	1
Access	Femoral	226	(100%)	81	(100%)	124	(100%)	21	(100%)	1
Any protection system	Distal	36	(15.9%)	12	(14.8%)	23	(18.5%)	1	(4.7%)	0.6702
	No	190	(84%)	69	(85.1%)	101	(81.4%)	20	(95.2%)	
Pre-dilatation	No	198	(87.6%)	71	(87.6%)	107	(86.2%)	20	(95.2%)	0.60705
	Yes	28	(12.3%)	10	(12.3%)	17	(13.7%)	1	(4.7%)	
Post-dilatation	Yes	226	(100%)	81	(100%)	124	(100%)	21	(100%)	1
Ulcerated	Yes	59	(26.1%)	23	(28.3%)	30	(24.1%)	6	(28.5%)	0.74269
Calcified	Yes	157	(69.4%)	59	(72.8%)	82	(66.1%)	16	(76.1%)	0.75776
Thrombotic	Yes	24	(10.6%)	8	(9.8%)	14	(11.2%)	2	(9.5%)	0.89662
Post-CEA restenosis	Yes	4	(1.7%)	1	(1.2%)	3	(2.4%)	0	(0%)	0.95965
Tortuosity	Absent	37	(16.3%)	10	(12.3%)	22	(17.7%)	5	(23.8%)	0.16177
	Low	70	(30.9%)	29	(35.8%)	32	(25.8%)	9	(42.8%)	
	Moderate	80	(35.3%)	27	(33.3%)	50	(40.3%)	3	(14.2%)	
	Severe	39	(17.2%)	15	(18.5%)	20	(16.1%)	4	(19%)	

In particular, in Table 5 relative values (normalized by the number of cases) of six categorical variables are presented (“Symptomatic,” “Ongoing stroke,” “Previous TIA < 24 h,” “Thrombotic,” “Ulcerated,” “Any protection system”). It can be observed that the distribution of the risk factors in the three groups is comparable. Moreover, Table 6 illustrates the continuous variable regarding stenosis improvement (“pre-operative and residual Stenosis Percentage”) and as it can be observed the distributions are homogeneous and no statistical differences are observed between the groups.

Consequently, the fact that the cerebrovascular risk factors (as reported in Tables 1 and 2 and shown in Tables 5 and 6) are balanced means that the results of the study are not influenced by any detectable bias and that the study classes are comparable.

### Procedural results and 24 h/48 h discharge results

Technical success was achieved in 100% of the cases. The average residual stenosis was 8.41%, with a standard deviation of ± 8.25, and no cases exceeded 30% of residual stenosis. Clinical success, defined as technical success without TIA or MACEs, was achieved in 98.7% (223 out of 226). One case (0.4%) experienced procedural complications and resulted in an ipsilateral major stroke. Additionally, during in-hospital observation (24 h), two additional TIAs (0.8%) were observed and fully recovered. There were no differences between the categories: technical success, complications, procedural success, residual stenosis, and in-hospital observations (TIA, major stroke, and major ipsilateral stroke at 24/48 h) (see Table 3). When analyzing the procedural MACEs distributed according to the CCA reference diameter classes, it was found that major ipsilateral stroke belonged to the class 2 group (diameter 6–7 mm). No patients with embolic protection filter presented MACEs.

**Table 3:** Procedural results and in-hospital observations, divided in CCA reference diameter class 1 [6–7) mm, class 2 [7–8) mm, and class 3 [8–9) mm

Title	Items	All	(All %)	Class1	(Class1%)	Class2	(Class2%)	Class3	(Class3%)	*p*-val
Procedure										
* N*		226		81		124		21		
Technical success	Yes	226	(100%)	81	(100%)	124	(100%)	21	(100%)	1
Complications	No	224	(99.1%)	80	(98.7%)	123	(99.1%)	21	(100%)	0.58159
	Yes	2	(0.8%)	1	(1.2%)	1	(0.8%)	0	(0%)	
Procedural success	Yes	226	(100%)	81	(100%)	124	(100%)	21	(100%)	1
Residual stenosis (percentage)	Mean (± std)	8.41	(± 8.25)	7.77	(± 7.57)	8.76	(± 8.81)	8.76	(± 7.56)	0.69137
	Median	7		7		7		10		
In-hospital observation (24 h)										
* N*		226		81		124		21	*N*	
TIA 24 h/48 h	Yes	2	(0.8%)	0	(0%)	2	(1.6%)	0	(0%)	0.54589
Major stroke 24 h/48 h	No	225	(99.5%)	81	(100%)	123	(99.1%)	21	(100%)	0.6701
Major ipsilateral stroke 24 h/48 h	Yes	1	(0.4%)	0	(0%)	1	(0.8%)	0	(0%)	

### Thirty-day follow-up results

In the follow-up at 30 days, it was found that a patient died after developing a hemorrhagic stroke on the contralateral side 21 days after the index procedure. A second subject treated for a tandem lesion involving large vessel occlusion of the cerebral medial artery (CMA) and internal carotid artery (ICA) experienced asymptomatic re-occlusion at 30 days (see Table 4).

**Table 4:** Follow-up at 30 days and 1 year, divided in CCA reference diameter class 1 [6–7) mm, class 2 [7–8) mm, and class 3 [8–9) mm

Title	Items	All	(All %)	Class1	(Class1%)	Class2	(Class2%)	Class3	(Class3%)	*p*-val
Follow-up (30 days)										
* N*		226		81		124		21		
New minor stroke 30 days	Yes	0	(0%)	0	(0%)	0	(0%)	0	(0%)	
New major stroke 30 days	Yes	1	(0.44%)	1	(1.2%)	0	(0%)	0	(0%)	0.1512
New death 30 days	Yes	1	(0.44%)	1	(1.2%)	0	(0%)	0	(0%)	0.1512
** New 30-day MACE**	**Yes**	**2**	**(0.88%)**	**2**	**(2.4%)**	**0**	**(0.0%)**	**0**	**(0%)**	
Accumulative minor stroke 30 days	Yes	0	(0%)	0	(0%)	0	(0%)	0	(0%)	
Accumulative major stroke 30 days	Yes	2	(0.88%)	1	(1.2%)	1	(1.2%)	0	(0%)	0.2314
Accumulative death 30 days	Yes	1	(0.44%)	1	(1.2%)	0	(0%)	0	(0%)	0.1512
** Accumulative 30-day MACE**	**Yes**	**3**	**(1.32%)**	**2**	**(2.4%)**	**1**	**(0.8%)**	**0**	**(0%)**	**0.2314**
Follow-up (1 year)										
Lost for follow-up		34	(15%)	18	(22.2%)	16	(12.9%)	0	(0%)	1
Follow-up patients at 1 year		192	(84.9%)	63	(77.7%)	108	(87%)	21	(100%)	
New 1-year MACE	Yes	0	(0%)	0	(0%)	0	(0%)	0	(0%)	
New 1-year MACE	Yes	0	(0%)	0	(0%)	0	(0%)	0	(0%)	
New minor stroke 1 year	Yes	0	(0%)	0	(0%)	0	(0%)	0	(0%)	
New major stroke 1 year	Yes	0	(0%)	0	(0%)	0	(0%)	0	(0%)	
Accumulative 1-year MACE	Yes	3	(1.5%)	2	(1.0%)	1	(0.5%)	0	(0%)	0.2314
Accumulative minor stroke 1 year	Yes	0	(0%)	0	(0%)	0	(0%)	0	(0%)	
Accumulative major stroke 1 year	Yes	2	(1.0%)	1	(0.5%)	1	(0.5%)	0	(0%)	0.2314
Accumulative death 1 year	Yes	1	(0.5%)	1	(0.5%)	0	(0%)	0	(0%)	0.1512
ICA patent 1 year	Yes	190	(99.0%)	61	(96.8%)	108	(100%)	21	(100%)	0.13381
ECA patent 1 year	Yes	191	(99.4%)	62	(98.4%)	108	(100%)	21	(100%)	0.13453

Bold values represent the sum of the previous lines

When analyzing the new 30-day MACEs distributed according to the CCA reference diameter classes, it was found that new major strokes and deaths belonged to the class 1 group (diameter 6–7 mm). The cumulative 30-day MACEs amount to 1.3% were distributed between the class 1 group (diameter 6–7 mm) and class 2 group (diameter 7–8 mm), with 2 and 1 cases, respectively.

### One-year follow-up results

From 30-day to 1-year follow-up, 34 patients were lost after repeated calls, mostly because of the COVID-19 pandemic. In the 192 patients group that completed the 1-year follow-up, no minor or major strokes were presented. The 1-year accumulative MACE ratio of the follow-up patient cohort was 1.5% and included two major strokes and one death, both of which occurred at 30 days. No new 1-year MACEs occurred in any classes between 30-day and 1-year follow-ups. Thus, the accumulative distribution according to the CCA reference diameter classes remains the same as the 30-day follow-up described in the previous paragraph.

Considering the patients with a 1-year Doppler ultrasound, ICA was patent in 99% of cases, and ECA was patent in 99.4% of cases.

## Discussion and conclusion

### Discussion

This study proposes the hypothesis that the one-size-fits-all strategy is feasible in the treatment of patients with carotid disease stenosis.

Current clinical practice suggests selecting a stent tailored to the CCA reference diameter. However, the key observation that initiated this research is that a nitinol CGuard 10-mm diameter stent has a working range applicable to vessel diameters from 4.5 to 9.5 mm, and it exhibits lower residual radial force compared to stents with smaller diameters. Therefore, the study evaluates the results of using the one-size-fits-all approach in a representative patient cohort divided into three reference diameter groups (or classes) with homogeneous risk factors and lesion characteristics.

In all patients, a 10-mm diameter stent was deployed, regardless of their reference CCA diameters. Procedural success and clinical outcomes were then compared across the groups to determine if any statistical differences existed between classes, thereby evaluating whether this strategy is equally safe and effective across different CCA diameters.

The results of this study corroborate the initial hypothesis. No significant differences between the groups were observed at the follow-up stages, including post-procedure, at 24–48 h/discharge, 30 days, and during the 1-year surveillance period.

The incidence of adverse events in this study is low, with a rate of 1.5% MACEs at 30 days, including two major strokes and one death without any further MACEs for the entire group at 1-year follow-up. Overall, the results across all stages were very positive compared to the performance reported in the CREST study [10], which indicated a 2.9% rate of death and stroke at 30 days.

The relative percentage of incidence in adverse events is similar across all three groups (see Tables 5 and 6). Consequently, the statistical analysis confirms that no differences are observed between the groups.

**Table 5:** Independent risk factors for post-procedural events distributed in the CCA reference diameter class 1 [6–7) mm, class 2 [7–8) mm, and class 3 [8–9) mm. In each row, “Symptomatic,” “Ongoing stroke,” and “Previous TIA < 24 h” are represented

Title	Items	All	(All %)	Class1 Abs	(Class1%) Rel	Class2	(Class2%)	Class3	(Class3%)	*p*-val
*N*		226		81		124		21		
Symptomatic for cerebral ischemia	Asymptomatic	43	(19%)	12	(14.8%)	25	(20.1%)	6	(28.5%)	0.13832
	Symptomatic	183	(80.9%)	69	(85.1%)	99	(79.8%)	15	(71.4%)	
Ongoing stroke	No	151	(66.9%)	55	(68%)	79	0.6371	17	(0.81%)	0.63221
	Yes	75	(33.1%)	26	(32%)	45	(36.2%)	4	(19%)	
Previous TIA < 24 h (transient ischemic attack)	No	132	(58.5%)	41	(50.7%)	78	(63%)	13	(62%)	0.12212
	Yes	94	(41.5%)	40	(49.3%)	46	(37%)	8	(38%)	
Thrombotic	No	202	(89.4%)	73	(90.2%)	110	(88.8%)	19	(90.5%)	0.89662
	Yes	24	(10.6%)	8	(9.8%)	14	(11.2%)	2	(9.5%)	
Ulcerated	No	167	(73.9%)	58	(71.7%)	94	(75.9%)	15	(71.5%)	0.74269
	Yes	59	(26.1%)	23	(28.3%)	30	(24.1%)	6	(28.5%)	
Any protection system	No	190	(84.1%)	69	(85.2%)	101	(81.5%)	20	(95.3%)	0.6702
	Distal	36	(15.9%)	12	(14.8%)	23	(18.5%)	1	(4.7%)

**Table 6:** Pre-procedural vs post-procedural residual stenosis percentage distributed in the CCA reference diameter class 1 [6–7) mm, class 2 [7–8) mm, and class 3 [8–9) mm

Title	Items	All	(All %)	Class1	(Class1%)	Class2	(Class2%)	Class3	(Class3%)	*p*-val
Stenosis pre (percentage)	Mean (± std)	78.99	(± 8.71)	79.49	(± 8.3)	79.4	(± 8.64)	74.61	(± 9.88)	0.53615
	Median	80		79		80		75		
Residual stenosis (percentage)	Mean (± std)	8.41	(± 8.25)	7.77	(± 7.57)	8.76	(± 8.81)	8.76	(± 7.56)	0.69137
	Median	7		7		7		10		

From the analysis of Tables 3 and 4, it can be seen that a few MACEs are reported in groups 1 and 2, while no MACEs are observed in group 3. However, it is not surprising that group 3 has no events, as the study’s MACE incidence of 1.5% corresponds to a 0.3% risk of having a MACE in the group of 21 patients.

Although the technical success rate was 100%, the potential risks of using an oversized stent in smaller vessels (such as strut malapposition, restenosis, or thrombus formation) should be carefully analyzed. Larger patient cohorts may be necessary to investigate these potential concerns.

The findings of the present study are consistent with those reported for both conventionally sized first- and second-generation carotid stents. Recent meta-analyses [10–12] have compared specific stent types, including first-generation (open- or closed-cell) and second-generation (mesh-covered) carotid stents, with results aligning with the outcome ranges reported above.

The 30-day clinical outcomes for first-generation carotid stents (open- or closed-cell, traditional size) have been extensively evaluated, with cumulative rates of death, stroke, and myocardial infarction ranging from 5.23% in the CREST Trial (2010) [13] to 2.27% in the PROTECT Trial (2012) [14].

Similarly, 30-day outcomes for second-generation mesh-covered carotid stents have shown cumulative rates between 4.80% in the SCAFFOLD Trial (2018) [15] and 3.18% in the ROADSTER 2 Trial (2024) [16].

At 1 year, first-generation carotid stents reported cumulative events (30-day death/stroke/MI plus ipsilateral stroke from 30 days to 1 year) ranging from 7.10% in the CREST Trial (2010) [13] to 3.80% in the ACT I Trial (2016). Second-generation stents reported 1-year cumulative events ranging from 5.86% in the COFIDENCE Trial (2024) [17] to 2.80% in the PERFORMANCE II Trial (2024) [18].

Regarding in-stent restenosis and re-occlusion at 1 year, first-generation stents demonstrate rates between 5.60% in the SPACE 2 Trial (2019) [19] and 5.20% in the CREST Trial (2010) [13]. For second-generation stents, rates range from 5.47% in the COFIDENCE Trial (2024) [18] to 4.10% in the SCAFFOLD Trial (2024) [20].

The advantage of the one-size-fits-all strategy lies in its ability to ensure optimal stent apposition to the vessel wall while minimizing residual radial force. Moreover, this approach facilitates a reduction in procedural time and hospital storage requirements.

### Limitations

The main limitation of this pilot study is that the one-size-fits-all strategy has not been directly compared with the standard procedure. A randomized study could confirm the benefits of the proposed strategy. Alternatively, larger studies could be considered to verify these effects findings. Other factors must be considered such as the low use of embolic protection systems and the high incidence of symptomatic patients when the clinical results are compared with the one obtained using the standard procedure.

Another limitation is the exclusive use of the CGuard stent. CGuard stents are not typically used for other arteries, such as the coronary or peripheral arteries. Their design is optimized for the carotid arteries, where embolic protection is crucial. Consequently, further investigation is required before directly applying the study’s results to other stent devices or different protective measures sleeve.

In this study, the use of embolic protection was left to the operator’s discretion. The pilot involved two highly experienced operators (over 10 years each). Despite its low usage, there was a low incidence of MACE. A larger multicenter study with more operators would allow to clarify the extent of operator dependence in these outcomes.

Due to COVID-19 restrictions, 15% of patients were lost to 1-year follow-up. While this rate is acceptable in clinical studies, it may slightly influence follow-up statistics.

Relevant details about the stent, such as Fig. 1 and the nitinol properties, were provided courtesy of InspireMD. This information was essential to accurately describe the specific mechanical behavior of the stent across different diameter sizes. It is essential to note that these data are not otherwise publicly available.

The patient data were provided in a blinded manner by clinical researchers who had no access to the final outcomes. Statistical analyses, table generation, and results interpretation were conducted by an independent researcher with a background in mathematics and computer science.

The interpretation of results and discussion was carried out collaboratively by all authors, ensuring objectivity and independence throughout the study.

### Conclusions

The results of this pilot study allow to conclude that the use of the one-size-fits-all strategy is feasible and safe at the 1-year follow-up. This is the first study that provides mid-term data on this strategy and may be relevant to informing clinical practice. Further analyses are required to compare the proposed strategy with other options, in order to elucidate their equivalence or superiority, which may include larger patient cohort studies and randomized comparisons.

## Supplementary Information

Supplementary Material 1.

## Data Availability

The data are property of the hospital and they cannot be shared.
