# Supplementary material for: One-size-fits-all strategy in carotid artery treatment using CGuard stent, feasibility and clinical pilot study
Source: CVIR Endovasc. 2025 Oct 16;8:86. doi: 10.1186/s42155-025-00601-7 (PMC12528512; doi:10.1186/s42155-025-00601-7)
Supplement: Supplementary file 1 — Supplementary Material 1. [file 42155_2025_601_MOESM1_ESM.pdf]

Supplementary material - Section 1 - Elastic and thermal shape recovery of nitinol

Figure SM-Figure 1 illustrates the elastic (left) and thermal (right) shape recovery of nitinol, showing how it regains its pre-defined shape upon force release below 37 °C and upon heating to body temperature.

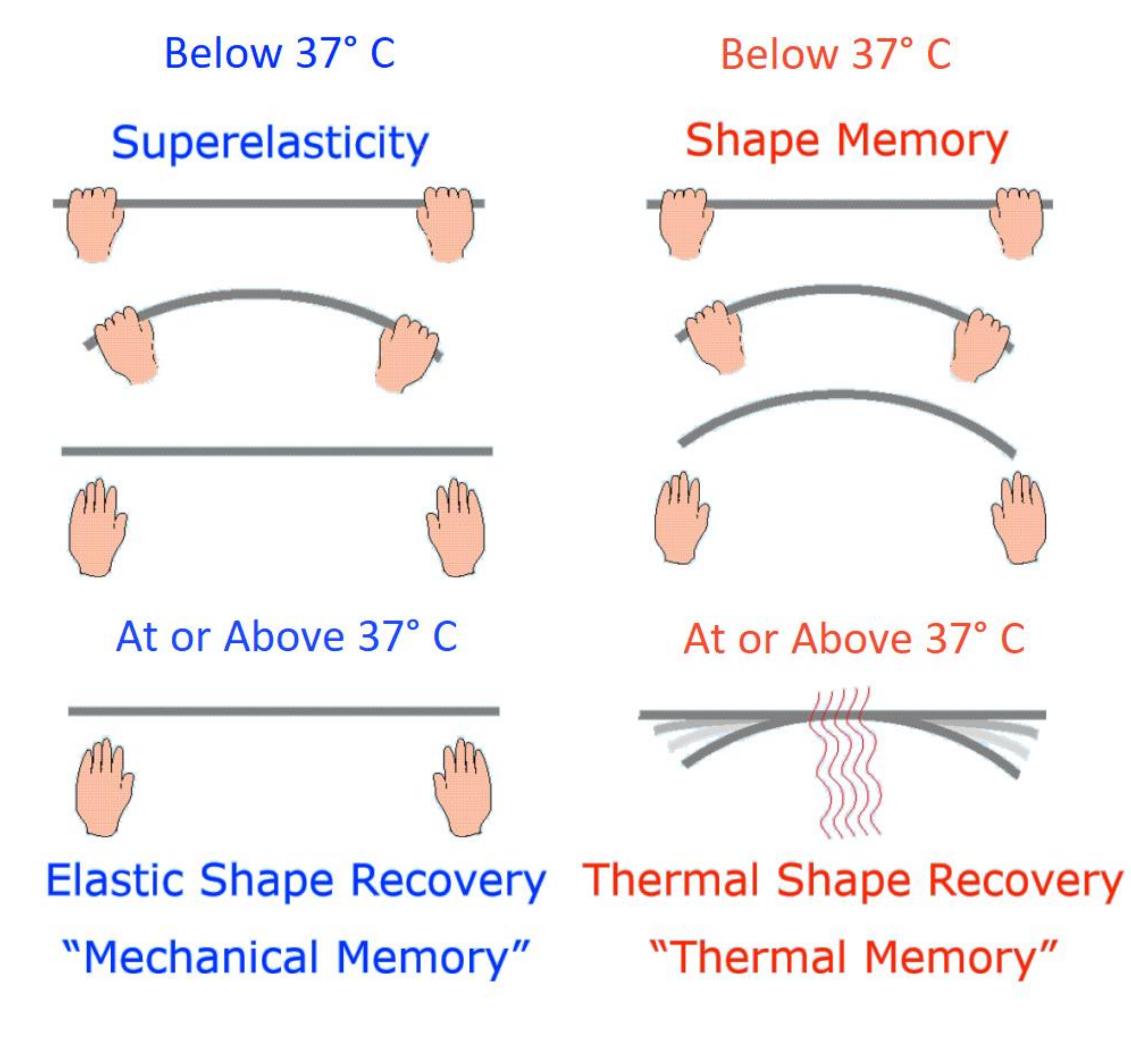

SM-Figure 1 scheme illustrating the elastic shape recovery, and the thermal shape recovery properties of a nitinol material. In the left picture it can be observed that the nitinol material (below 37° C) return to the pre-defined shape, when the force is released. Whereas on the right picture it can be seen that the nitinol doesn't return to the original shape when the force is released, however, when the body temperature is reached (at or above 37° C) the material return to its pre-defined shape.

Supplementary material - Section 2 Illustration of a CGuard nitinol stent

SM-Figure 2 illustrates (a) the CGuard nitinol stent with an open-cell design and 165-micron PET mesh, and (b) the angiographic view of the implanted stent.

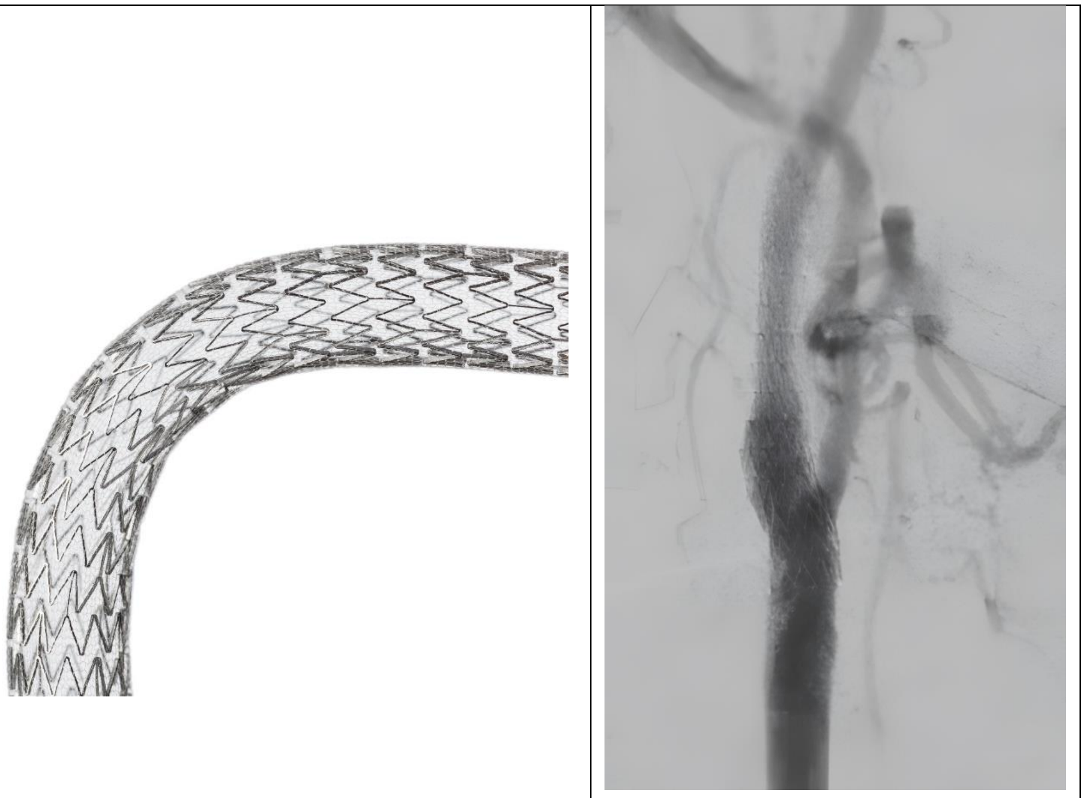

(a)	(b)

SM-Figure 2 (left - a) Picture of the CGuard nitinol stent with open cell design with a PET mesh with 165 micron of porous size. (Right - b) Angiographic view of the implanted stent

Supplementary material - Section 3 Influence of risk factors

In order to further analyze the influence of the Independent risk factor over the post-procedural events of the study, we stratified the patient population in five sub-groups:
-“prevMACE” included: “Ongoing Stroke”, “Minor Stroke”, “Major Stroke” risk factors of Table 2 and presented in Figure 2
-“Shape” included: “Thrombotic”, “Ulcerated” and “Tortuosity” risk factors of Table 2
-“OtherRF” included: “Symptomatic for Isquemia”, “Previous TIA <24h”, “Any Protection System” risk factors of Table 2
-“Control” included patients presenting none of the previous risk factors.
-“All” included all the whole study population

The categories are chosen according to the following criteria: “prevMACE” includes patients who had previous cardiovascular events before the intervention. “Shape” refers to those with complex anatomies. “OtherRF” covers the remaining independent risk factors shown in Figure 2. Finally, the “Control” group includes patients with none of the above risk factors.

As it can be observed in Table 7, the post-procedural events of the whole population are distributed over the five patient sub-groups.

In particular, the “Complications” post-procedural events present in class1 and class2 stent sizes distributed in “Shape” , “prevMACE” and “OtherRF” groups. The two “TIA” split in “prevMACE” and “Shape” categories. The “Major Stroke 24h/48h” and “Stroke Ipsilateral” post-procedural events, appear in a patient having both “prevMACE” and “OtherRF”. The three “30d-MACE” post-procedural events occurred in class1 and class2 stents splits into “prevMACE” and “OtherRF” and specifically two of them belongs to two different classes of “OtherRF”. The “Major stroke 30d” post-procedural event is related to patient with complex “Shape”. And finally the “Death 30d” post-procedural event happened in a patient with complex “Shape” having also “OtherRF”.

In conclusion, Table 7 shows that post-procedural events are not specifically correlated with any particular risk factor or stent class (size), but are instead distributed across all categories and classes. Their higher occurrence in stent Classes 1 and 2 is expected, given the larger patient population in these groups.

Table 7 Distribution of the post-procedural events of the whole population over the five patients sub-groups (“All”, “Control”,“prevMACE”, “Shape”, “OtherRF”)

Title		Items	All	(All)	Class1	(Class1)	Class2	(Class2)	Class3	(Class3)

Complications	All	yes	2	(0.8 %)	1	(1.2 %)	1	(0.8 %)	0	(0 %)
	Control	no	98	(100 %)	34	(100 %)	55	(100 %)	9	(100 %)
	prevMACE	yes	1	(1.2 %)	0	(0 %)	1	(2.1 %)	0	(0 %)
	Shape	yes	1	(1 %)	1	(2.6 %)	0	(0 %)	0	(0 %)
	OtherRF	yes	1	(0.5 %)	0	(0 %)	1	(0.9 %)	0	(0 %)
TIA 24h/48h	All	yes	2	(0.8 %)	0	(0 %)	2	(1.6 %)	0	(0 %)
	control	no	98	(100 %)	34	(100 %)	55	(100 %)	9	(100 %)
	prevMACE	yes	1	(1.2 %)	0	(0 %)	1	(2.1 %)	0	(0 %)
	Shape	yes	2	(2 %)	0	(0 %)	2	(4 %)	0	(0 %)
	OtherRF	yes	2	(1 %)	0	(0 %)	2	(1.9 %)	0	(0 %)
Minor Stroke 24h/48h	All	no	226	(100 %)	81	(100 %)	124	(100 %)	21	(100 %)
	Control	no	98	(100 %)	34	(100 %)	55	(100 %)	9	(100 %)
	prevMACE	no	79	(100 %)	27	(100 %)	47	(100 %)	5	(100 %)
	Shape	no	97	(100 %)	38	(100 %)	49	(100 %)	10	(100 %)
	OtherRF	no	189	(100 %)	72	(100 %)	102	(100 %)	15	(100 %)
Major Stroke 24h/48h	All	yes	1	(0.4 %)	0	(0 %)	1	(0.8 %)	0	(0 %)
	Control	no	98	(100 %)	34	(100 %)	55	(100 %)	9	(100 %)
	prevMACE	yes	1	(1.2 %)	0	(0 %)	1	(2.1 %)	0	(0 %)
	Shape	no	97	(100 %)	38	(100 %)	49	(100 %)	10	(100 %)
	OtherRF	yes	1	(0.5 %)	0	(0 %)	1	(0.9 %)	0	(0 %)
Stroke Ipsilateral	All	yes	1	(0.4 %)	0	(0 %)	1	(0.8 %)	0	(0 %)
	Control	no	98	(100 %)	34	(100 %)	55	(100 %)	9	(100 %)
	prevMACE	yes	1	(1.2 %)	0	(0 %)	1	(2.1 %)	0	(0 %)
	Shape	no	97	(100 %)	38	(100 %)	49	(100 %)	10	(100 %)
	OtherRF	yes	1	(0.5 %)	0	(0 %)	1	(0.9 %)	0	(0 %)
Death	ALL	no	226	(100 %)	81	(100 %)	124	(100 %)	21	(100 %)
	Control	no	98	(100 %)	34	(100 %)	55	(100 %)	9	(100 %)
	prevMACE	no	79	(100 %)	27	(100 %)	47	(100 %)	5	(100 %)
	Shape	no	97	(100 %)	38	(100 %)	49	(100 %)	10	(100 %)
	OtherRF	no	189	(100 %)	72	(100 %)	102	(100 %)	15	(100 %)
30d-MACE	All	yes	3	(1.3 %)	2	(2.4 %)	1	(0.8 %)	0	(0 %)
	Control	no	98	(100 %)	34	(100 %)	55	(100 %)	9	(100 %)

	prevMACE	yes	1	(1.2 %)	0	(0 %)	1	(2.1 %)	0	(0 %)
	Shape	no	95	(97.9 %)	36	(94.7 %)	49	(100 %)	10	(100 %)
	OtherRF	yes	2	(1 %)	1	(1.3 %)	1	(0.9 %)	0	(0 %)
TIA 30d	All	no	226	(100 %)	81	(100 %)	124	(100 %)	21	(100 %)
	Control	no	98	(100 %)	34	(100 %)	55	(100 %)	9	(100 %)
	prevMACE	no	79	(100 %)	27	(100 %)	47	(100 %)	5	(100 %)
	Shape	no	97	(100 %)	38	(100 %)	49	(100 %)	10	(100 %)
	OtherRF	no	189	(100 %)	72	(100 %)	102	(100 %)	15	(100 %)
Minor stroke 30d	All	no	226	(100 %)	81	(100 %)	124	(100 %)	21	(100 %)
	Control	no	98	(100 %)	34	(100 %)	55	(100 %)	9	(100 %)
	prevMACE	no	79	(100 %)	27	(100 %)	47	(100 %)	5	(100 %)
	Shape	no	97	(100 %)	38	(100 %)	49	(100 %)	10	(100 %)
	OtherRF	no	189	(100 %)	72	(100 %)	102	(100 %)	15	(100 %)
Major stroke 30d	All	yes	1	(0.4 %)	1	(1.2 %)	0	(0 %)	0	(0 %)
	Control	no	98	(100 %)	34	(100 %)	55	(100 %)	9	(100 %)
	prevMACE	no	79	(100 %)	27	(100 %)	47	(100 %)	5	(100 %)
	Shape	yes	1	(1 %)	1	(2.6 %)	0	(0 %)	0	(0 %)
	OtherRF	no	189	(100 %)	72	(100 %)	102	(100 %)	15	(100 %)
Death 30d	All	yes	1	(0.4 %)	1	(1.2 %)	0	(0 %)	0	(0 %)
	Control	no	98	(100 %)	34	(100 %)	55	(100 %)	9	(100 %)
	prevMACE	no	79	(100 %)	27	(100 %)	47	(100 %)	5	(100 %)
	Shape	yes	1	(1 %)	1	(2.6 %)	0	(0 %)	0	(0 %)
	OtherRF	yes	1	(0.5 %)	1	(1.3 %)	0	(0 %)	0	(0 %)
Symptomatic	ALL	yes	183	(80.9 %)	69	(85.1 %)	99	(79.8 %)	15	(71.4 %)
	Control	yes	71	(72.4 %)	28	(82.3 %)	38	(69 %)	5	(55.5 %)
	prevMACE	yes	79	(100 %)	27	(100 %)	47	(100 %)	5	(100 %)
	Shape	yes	80	(82.4 %)	32	(84.2 %)	40	(81.6 %)	8	(80 %)
	OtherRF	yes	183	(96.8 %)	69	(95.8 %)	99	(97 %)	15	(100 %)

Supplementary material - Section 4 Risk factor analysis

Symptomatic patients undergoing CAS represent a known post-procedural risk group for MACEs. The complete list of cerebrovascular risk factors is presented in Table 1 according to class distributions. As shown in Table 1, no statistically significant differences were found among the risk variables, as indicated by their non-significant p-values, ensuring that the groups are balanced is crucial to discard any bias in clinical outcome differences due to variations in the patient population. Therefore, from Table 2 and Table 3 the analysis of independent risk factors [3] is performed quantitatively in in Table 5 and 6, and qualitatively in SM-Figures 3 and SM-Figures 4.

In particular, in SM-Figure 3 the relative values of six categorical variables are presented (“Symptomatic”, “Ongoing stroke”, “Previous TIA <24h”, “Thrombotic”, “Ulcerated”, “Any protection system”) which were presented in Table 5. Moreover, SM-Figure 4 illustrates the continuous variable regarding stenosis improvement (“pre-operative and residual Stenosis Percentage”) which were presented in Table 6, by means of a boxplot.

When the absolute values of SM-Figure 4, it can be observed that the distribution of the risk factors in the three groups is comparable. Considering Figure 3 (a and b), the boxplot quartiles are overlapped, indicating that the distribution is homogeneous.

Consequently, the fact that the cerebrovascular risk factors (as reported in Table 5 and Table 6, and shown in SM-Figure 3 and SM-Figure 4) are balanced means that the results of the study are not influenced by any detectable bias and that the study classes are comparable.

Symptomatic for Cerebral isquemia	Ongoing stroke
p-val = 0.13832 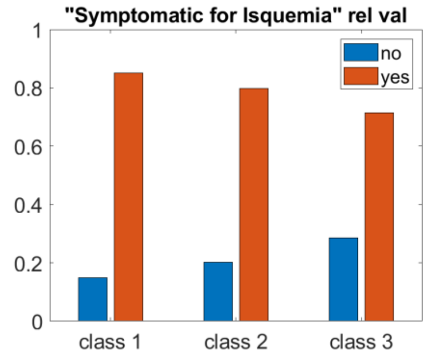	p-val =0.63221 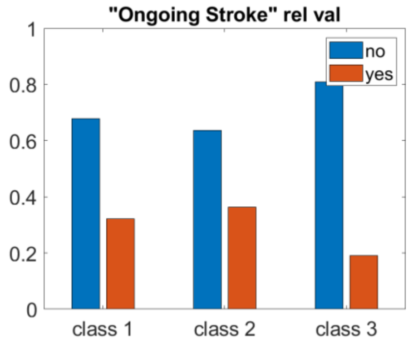

Previous TIA <24h	Thrombotic

p-val =0.12212

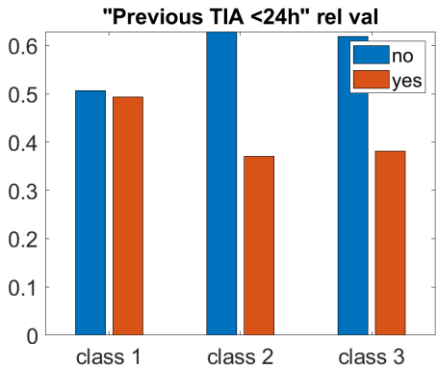

p-val = 0.89662

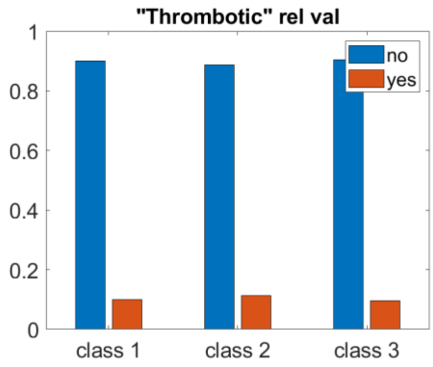

Ulcerated	Any protection system

p-val = 0.74269

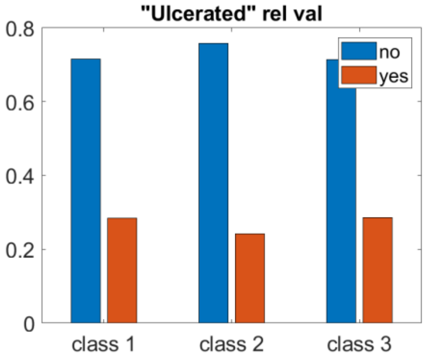

p-val = 0.6702

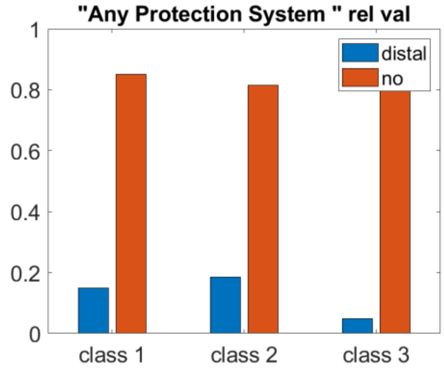

SM-Figure 3. Independent risk factors for post-procedural events distributed in the CCA reference diameter Class 1 [6-7) mm, Class 2 [7-8) mm and Class 3 [8-9 mm). In each row, "Symptomatic", "Ongoing Stroke" and "Previous TIA <24h" are represented.

Stenosis pre (percentage)	*Residual stenosis (percentage)*

p-val = 0.053615	p-val = 0.69137
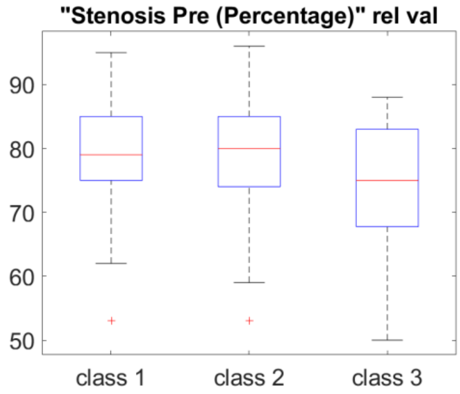	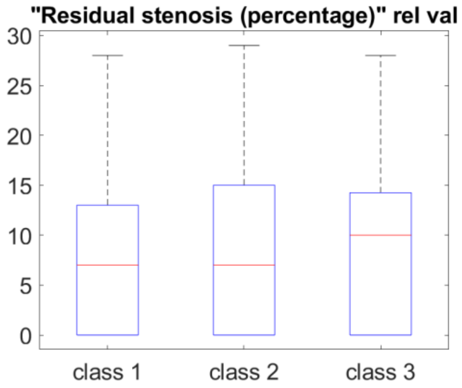
a	*b*

SM-Figure 4. Pre-procedural (column a) vs post-procedural (column b) residual stenosis percentage distributed in the CCA reference diameter Class 1 [6-7) mm, Class 2 [7-8) mm and Class 3 [8-9 mm).
